# Supplementary material for: Fine-mapping and molecular characterisation of primary sclerosing cholangitis genetic risk loci
Source: Nat Commun. 2024 Nov 6;15:9594. doi: 10.1038/s41467-024-53602-w (PMC11541731; doi:10.1038/s41467-024-53602-w)
Supplement: Supplementary file 1 — Supplementary Information [file 41467_2024_53602_MOESM1_ESM.pdf]

Fine-mapping and molecular characterisation of primary sclerosing cholangitis genetic risk loci

Short title: Genetic characterisation of PSC

Authors: Elizabeth C. Goode[1,2,3], Laura Fachal[1], Nikolaos Panousis[1], Loukas Moutsianas[1], Rebecca E. McIntyre[1], Benjamin Yu Hang Bai[1,2], Norihito Kawasaki[4], Alexandra Wittmann[4], Tim Raine[2], Simon M Rushbrook[3,5], Carl A. Anderson[1*].

Affiliations: [1]Wellcome Sanger Institute, Hinxton, Cambridge, UK, [2]University of Cambridge, Cambridge, UK, [3]Norfolk and Norwich University Hospital, Norwich, UK, [4]Quadrum Institute, Norwich, UK, [5]Norwich Medical School, University of East Anglia, Norwich, UK.

Supplementary Information

Supplementary Tables

Supplementary Table 1: Characteristics of the PSC-specific eQTL map study cohort according to disease group.

	PSC-UC (n=42)	UC (n=32)
**Gender (% Male)**	81	69
**Mean Age (Range)**	50 (17-86)	52 (38-75)
**UDCA use (%)**	71	0
**5-ASA use (%)**	66	90
**Azathioprine use (%)**	10	21

Supplementary Table 2: Results of colocalisation between T-cell eQTLs mapped in PSC-UC patients and GWAS risk loci for Ulcerative colitis (UC), Crohn's disease (CD), Rheumatoid arthritis (RhA) and Type 1 Diabetes (T1DM). Beta and two-sided test p-values were estimated through linear regression via QTLtools.

Chr	GWAS SNP	Disease	eGene	Cell type	PP4	eQTL Beta	eQTL p-val
1	rs3180018	UC	*GBAP1*	T-regulatory	0.91	-0.74	3.88E-04
				T-memory	0.98	-1.01	1.30E-10
				CD4+CCR9-	0.98	-0.92	2.01E-07
				CD4+CCR9+	0.98	-0.91	7.66E-07
		UC	*THBS3*	CD4+CCR9-	0.98	0.88	5.59E-07
1	rs2317230	RhA	*FCRL3*	CD8+CCR9-	0.93	0.82	3.29E-04
5	rs7731626	RhA	*IL6ST*	T-regulatory	0.97	-0.86	2.08E-04
				T-memory	0.90	-0.81	6.16E-04
		RhA	*ANKRD55*	T-memory	1.00	-1.00	5.94E-07
				CD4+CCR9-	1.00	-0.95	9.63E-06
				CD4+CCR9+	0.86	-0.86	1.20E-03
7	rs4728142	UC	*IRF5*	T-memory	0.86	0.77	4.71E-05
11	rs663743	PSC	*AP003774.1*	T-regulatory	0.99	0.98	7.27E-06
				T-memory	0.95	1.07	1.35E-07
				CD4+CCR9-	0.95	0.96	1.83E-05
11	rs968567	RhA	*FADS1*	T-regulatory	0.89	1.51	1.38E-07
		RhA	*FADS2*	T-regulatory	0.98	1.60	2.01E-09
				T-memory	1.00	1.58	2.23E-09
				CD4+CCR9-	0.99	1.58	5.40E-09
				CD4+CCR9+	0.98	1.47	2.82E-07
				CD8+CCR9-	0.96	1.56	9.58E-09
				CD8+CCR9+	0.95	1.46	4.22E-07
12	rs4760341	T1DM	*SUOX*	T-regulatory	0.80	-0.71	1.06E-03
14	rs941576	T1DM	*WARS*	T-regulatory	0.85	1.07	3.90E-05
				T-memory	0.93	1.19	1.46E-07
				CD4+CCR9-	0.80	1.35	2.41E-08
				CD8+CCR9-	0.97	-1.03	1.37E-05
19	rs4802307	CD	*PPP5C*	T-memory	0.85	-0.92	1.63E-06
				CD4+CCR9-	0.86	-0.99	2.38E-08
				CD8+CCR9-	0.83	-0.82	1.48E-04
21	rs1893592	PSC	*UBASH3A*	T-mempry	0.91	0.93	4.83E-04
22	rs909685	RhA	*SYNGR1*	CD8+CCR9+	0.98	1.14	7.15E-04

UC; Ulcerative colitis, RhA; rheumatoid arthritis, T1DM; Type 1 diabetes mellitus, CD; Crohn's disease, PSC; Primary sclerosing cholangitis.

Supplementary Figures

Supplementary Figure 1: Enrichment analyses results.

The y axis shows the enrichment (Prop.h2/Prop.SNPs) per cell type, whereas the y axis indicates the cell types. Results have been coloured by cell type, grouping different cell types in a broader category (ie, Th17 precursors, Memory Teff etc into T-cell). Panel a): open chromatin regions (ATAC-seq) from immune cell types under resting (_U) and stimulated (_S) conditions[1]. Panel b): cis candidate regulatory elements (cCRE) inferred from single cell ATAC-seq from 30 adult tissues[2]. qvalues were estimated from the LDSC two sided test p-values to nominate significant results (qvalue < 0.05), labelled with an asterisk in the plot. Bars represent the enrichment standard error.

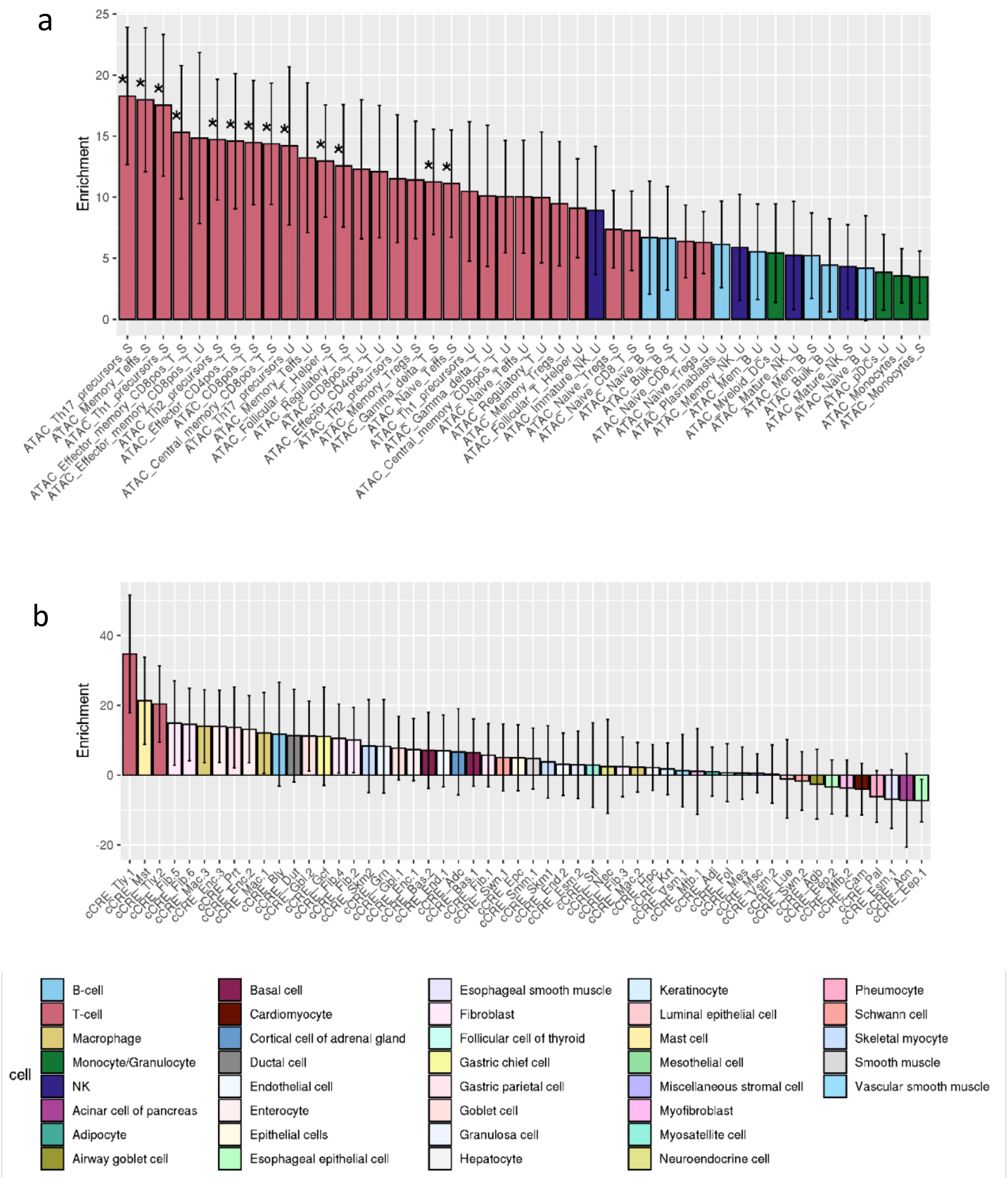

Supplementary Figure 2: Distance to between eQTL and transcription start site

Distance from transcription start site (TSS) for each significant eQTL (coloured red for those less than 5% FDR) per cell type. Panel a); T-regulatory cells, b); T-memory cells, c); CD4+CCR9- T-cells, d); CD4+CCR9+ T-cells, e); CD8+CCR9- T-cells, f); CD8+CCR9+T-cells.

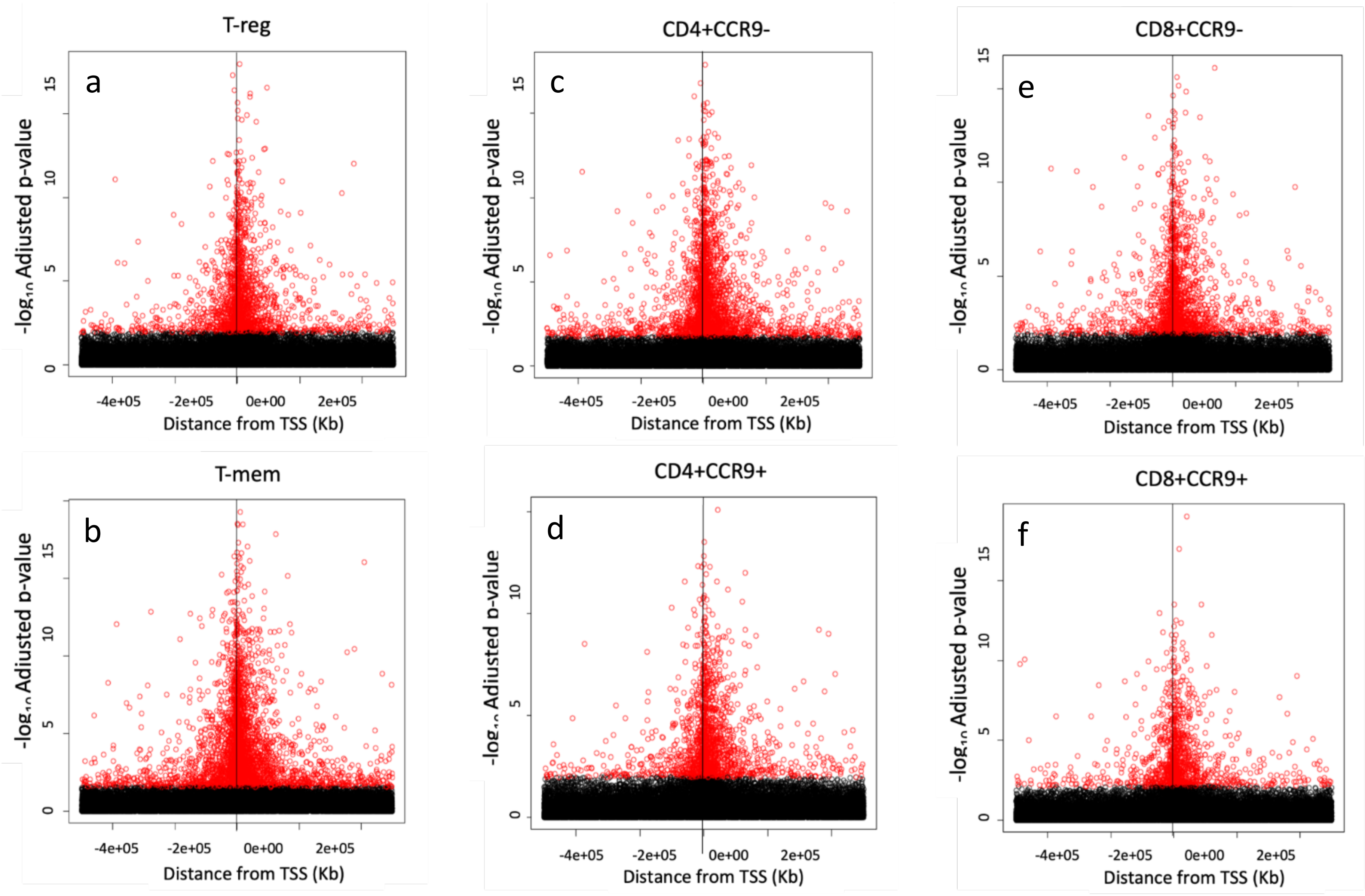

Supplementary Figure 3: Posterior z-scores of significant eGenes as identified by mashR

Posterior z-scores of significant eGenes as identified by mashR (lfdr <0.05 in at least one PSC T cell subtype; N = 10,459), grouped by (i) significant eGenes shared across all cell types (panel a), N = 9,176); (ii), significant eGenes in two or more cell types, but not in all cell types all (panel b), N = 794); and (iii) those condition specific eGenes, only significant in one cell type (panel c), N = 489).

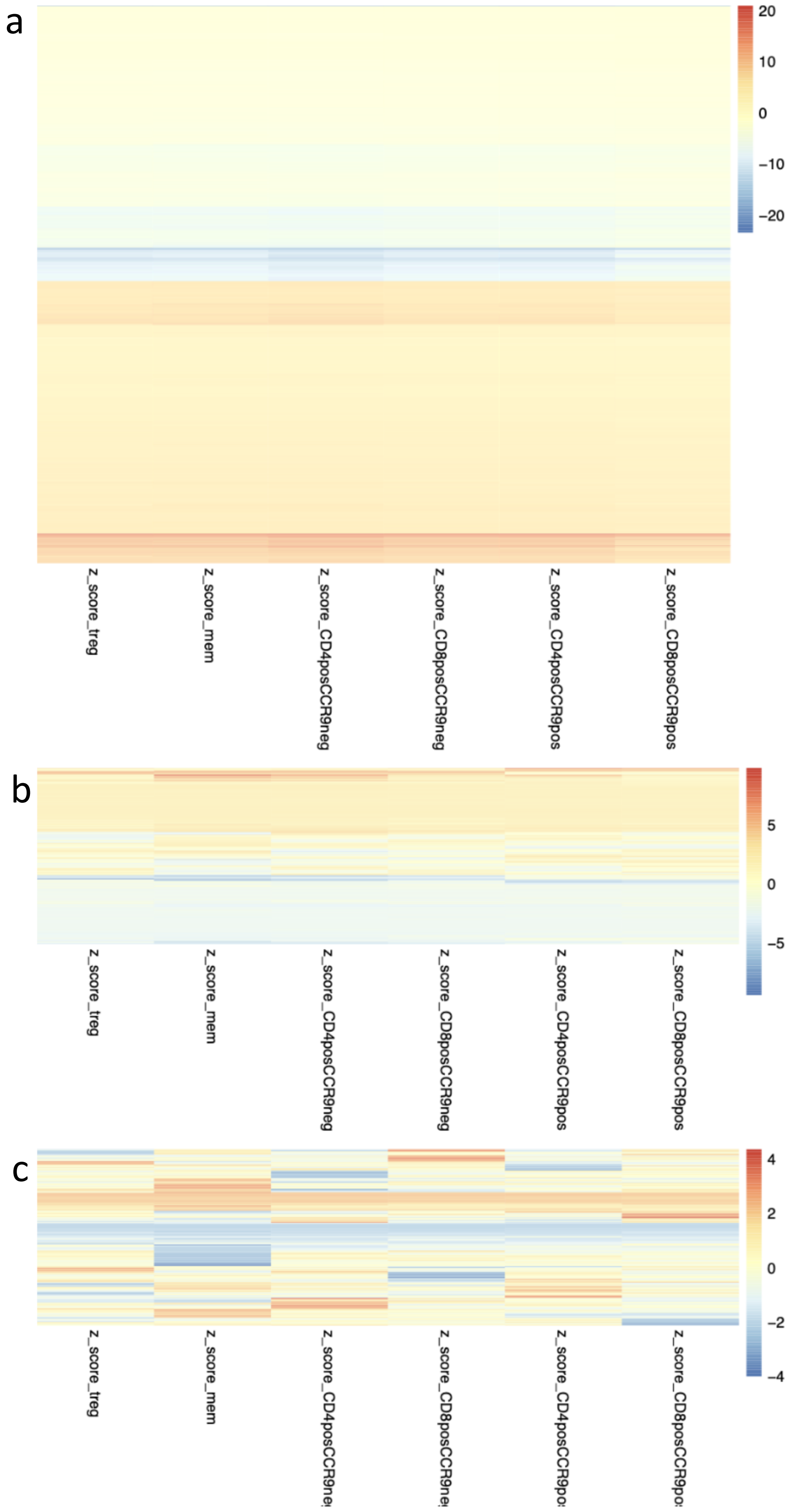

Supplementary Figure 4: Replication of significant eGenes in each PSC T-cell subtype

Proportion of significant eGenes in each PSC T cell subtypes (eQTL qvalue < 0.05; estimated from the two-sided p-values derived by QTLtools) replicated in external datasets (pi1)

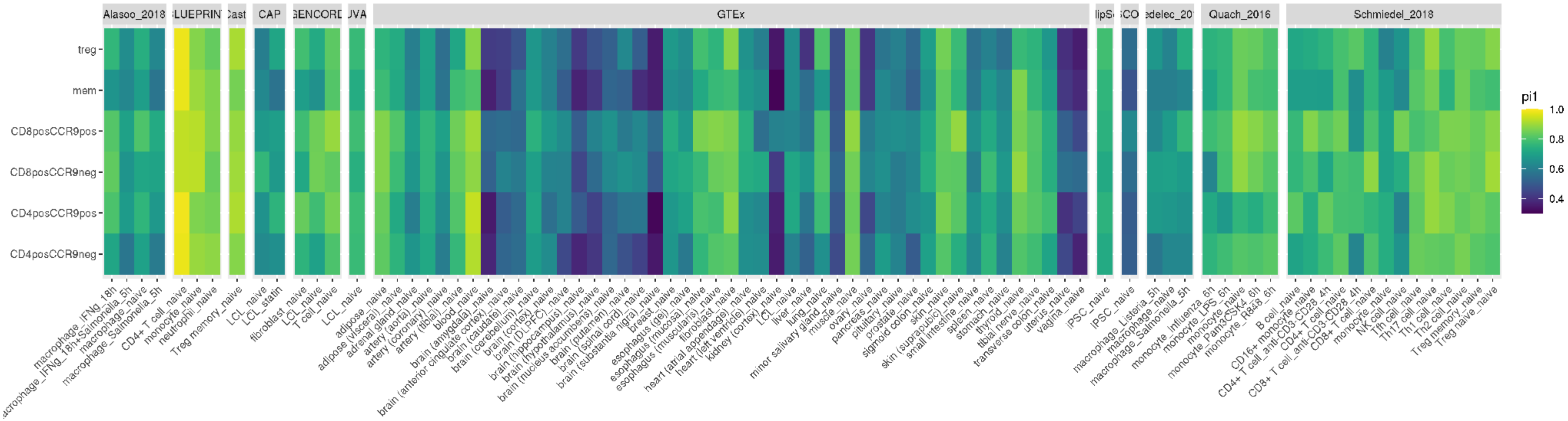

Supplementary Figure 5: Colocalisation results for CCDC88B Chr11 region

CCDC88B Chr11: regional association plot for a) PSC GWAS data (NCases= 4,796; NCtr= 19,955) and b) colocalising (PP4≥ 0.8) eQTL data for CCDC88B in monocytes (N = 194). The most-likely causal variant (rs663743 PP=0.85) is shown in purple. LD information is calculated from PSC GWAS data[3]

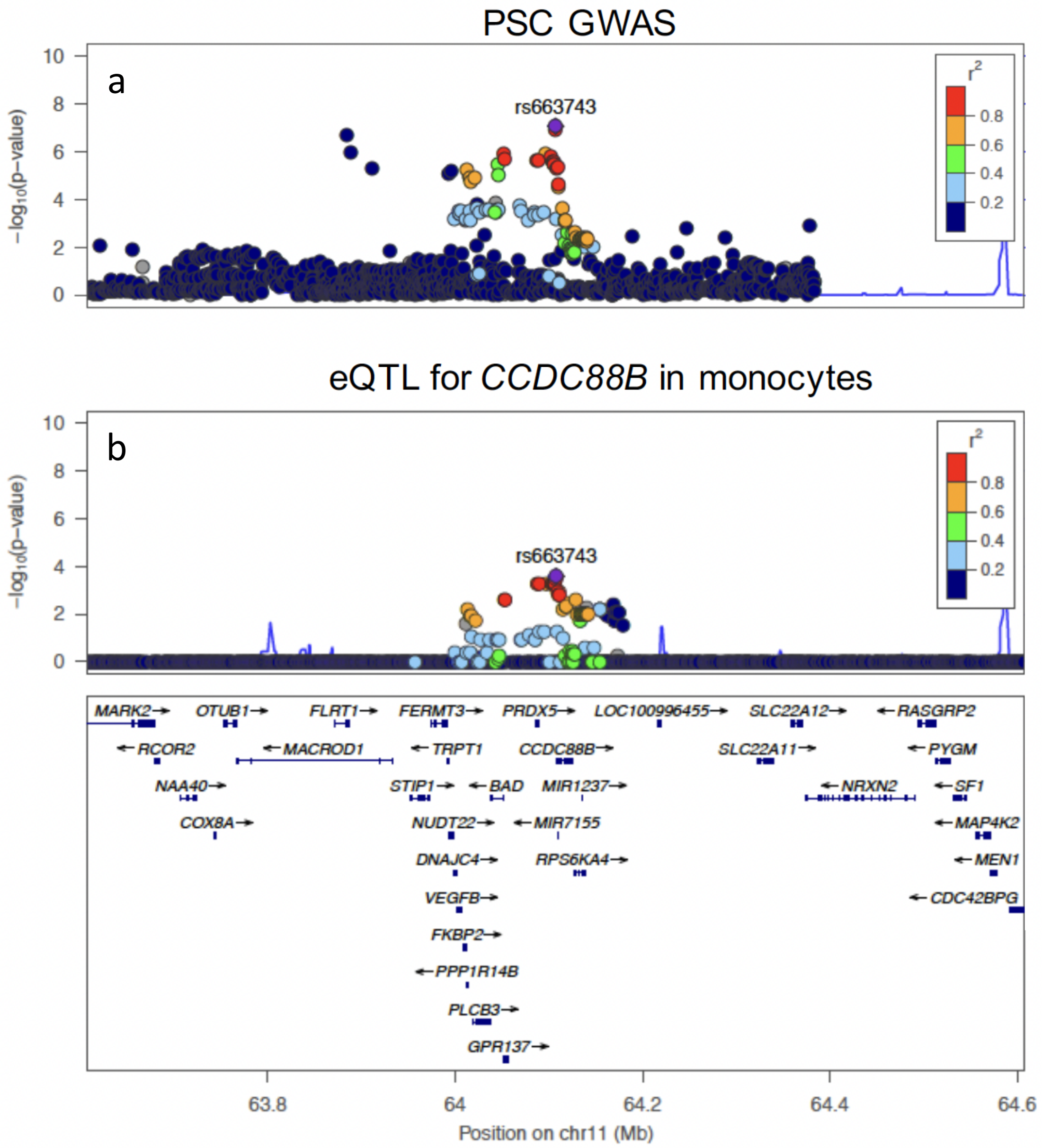

Supplementary Figure 6: FACS gating strategy for PSC eQTL maps

FACS gating strategy used for PSC specific T-cell separation with sequential sorting from left to right. Panel a); Forward versus side scatter, b); forward scatter height versus width, c); CD3+ versus forward scatter height, d); CD3 versus CD62L, e); CD45RO versus CD3+, f); CD4 versus CD8, g); CCR9 versus CD4.

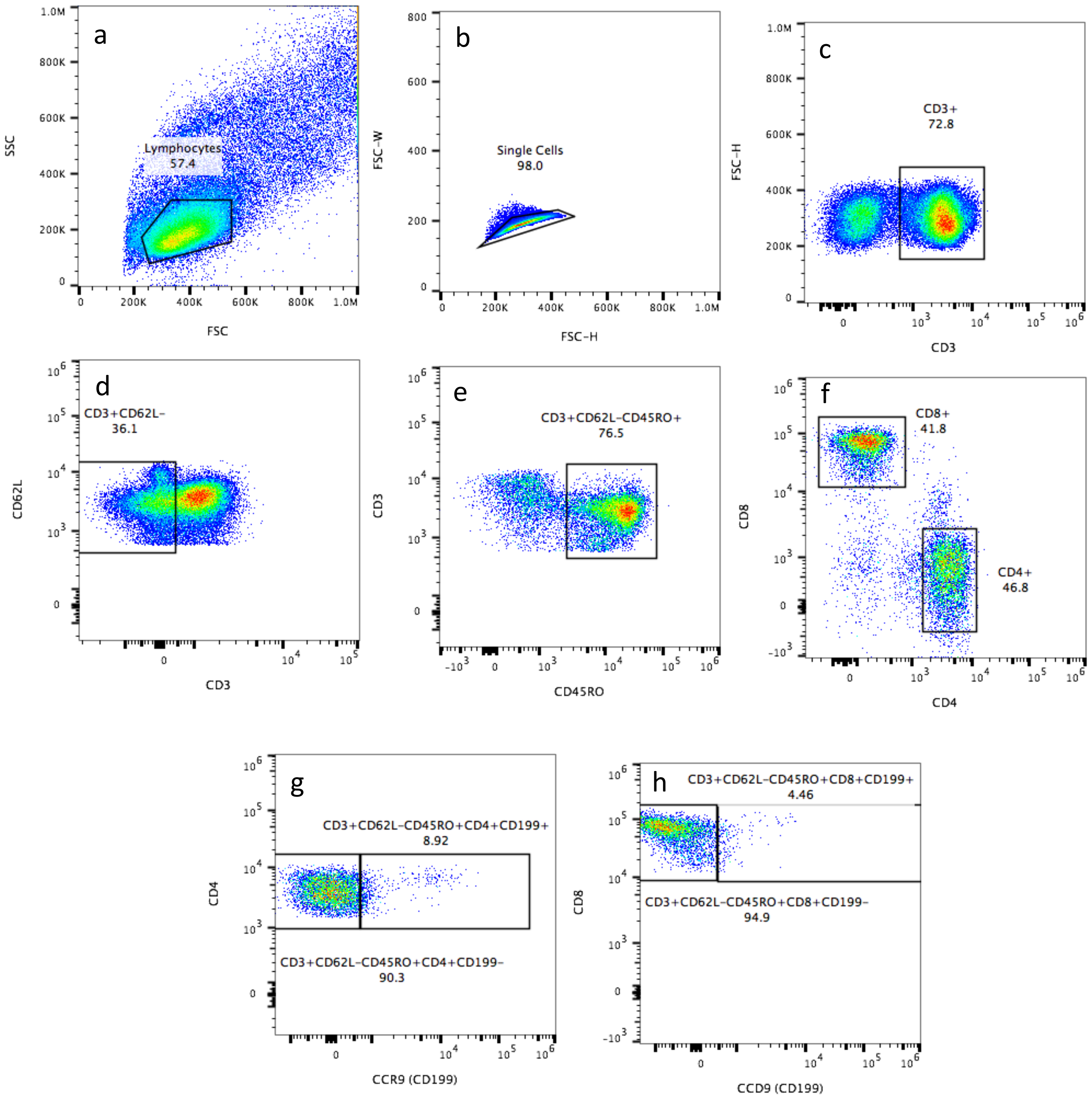

Supplementary Figure 7: Principal component analysis of study samples compared to 1000 Genomes.
Principal component analysis of study samples compared to 1000 Genomes samples of known ethnicity using a pruned set of 62,805 independent variants with an r2<0.2 and MAF>0.01. Panel a) shows PSC samples compared to all ethnicities, and panel b) shows European subpopulations. Three individuals were of Southern European/Iberian ethnicity, highlighted on the Figure. All samples from individuals of Northern and Southern European ethnicity were retained for further analysis.

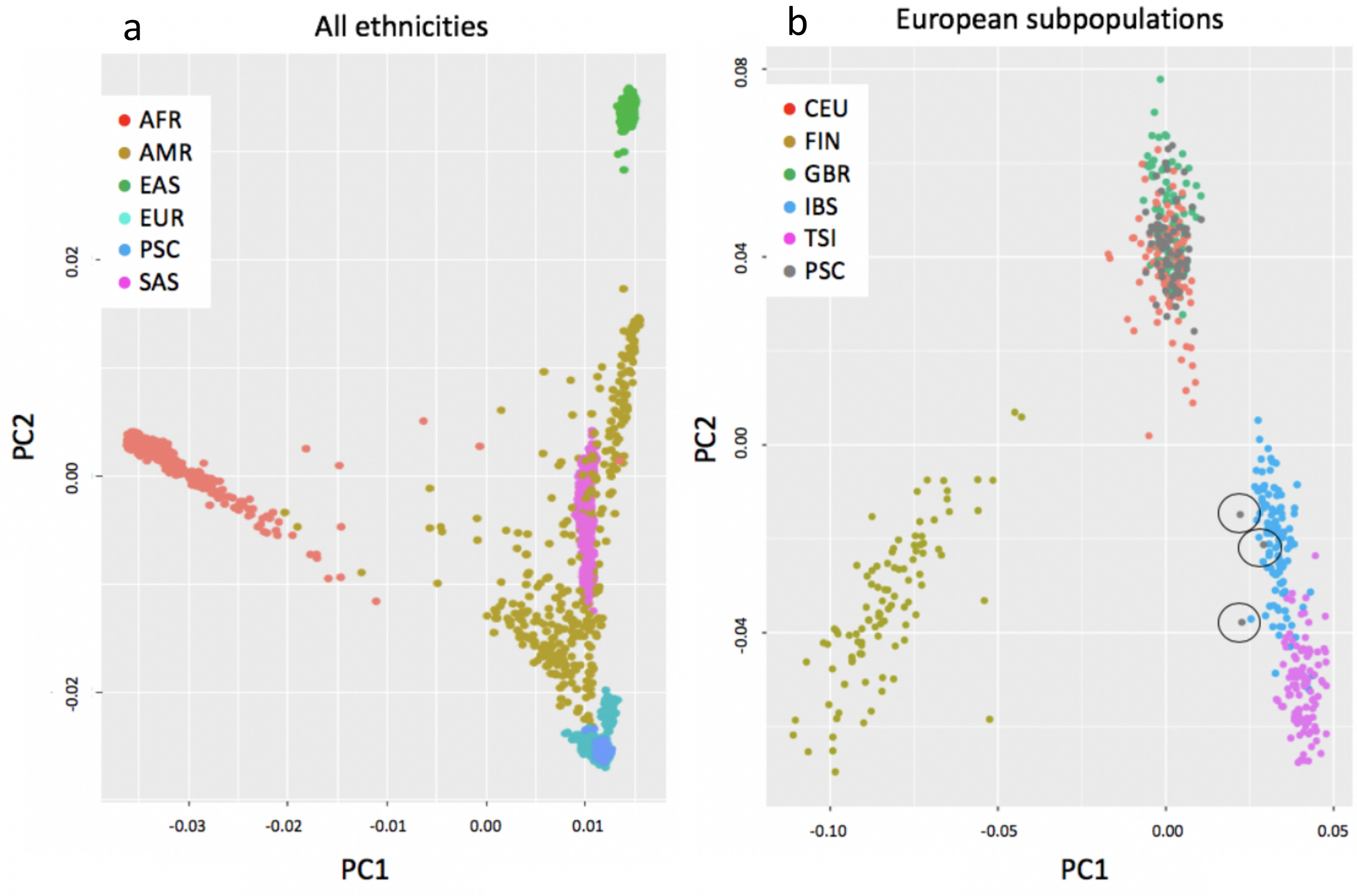

Supplementary References

1. Calderon D, Nguyen MLT, Mezger A, et al. Landscape of stimulation-responsive chromatin across diverse human immune cells. Nat Genet 2019;51:1494-1505.

2. Zhang K, Hocker JD, Miller M, et al. A single-cell atlas of chromatin accessibility in the human genome. Cell 2021;184:5985-6001 e19.

3. Maurano MT, Humbert R, Rynes E, et al. Systematic localization of common disease-associated variation in regulatory DNA. Science 2012;337:1190-5
