# Supplementary material for: Fine-mapping and molecular characterisation of primary sclerosing cholangitis genetic risk loci
Source: Nat Commun. 2024 Nov 6;15:9594. doi: 10.1038/s41467-024-53602-w (PMC11541731; doi:10.1038/s41467-024-53602-w)
Supplement: Supplementary file 3 — Description of additional supplementary files [file 41467_2024_53602_MOESM3_ESM.pdf]

Description of Additional Supplementary Files

Supplementary Data 1 Characteristics of datasets included in colocalisation analysis

Supplementary Data 2 Colocalisation of PSC risk loci with molecular QTLs

Supplementary Data 3 FACS Gating strategy
