# Supplementary material for: Fine-mapping and molecular characterisation of primary sclerosing cholangitis genetic risk loci
Source: Nat Commun. 2024 Nov 6;15:9594. doi: 10.1038/s41467-024-53602-w (PMC11541731; doi:10.1038/s41467-024-53602-w)
Supplement: Supplementary file 7 — Reporting summary [file 41467_2024_53602_MOESM7_ESM.pdf]

nature portfolio

Corresponding author(s): Dr Carl A Anderson

Last updated by author(s): 1st July 2024

Reporting Summary

Nature Portfolio wishes to improve the reproducibility of the work that we publish. This form provides structure for consistency and transparency in reporting. For further information on Nature Portfolio policies, see our Editorial Policies and the Editorial Policy Checklist.

Please do not complete any field with "not applicable" or n/a. Refer to the help text for what text to use if an item is not relevant to your study.
For final submission: please carefully check your responses for accuracy; you will not be able to make changes later.

Statistics

For all statistical analyses, confirm that the following items are present in the figure legend, table legend, main text, or Methods section.

n/a	Confirmed	
☐	☒	The exact sample size (*n*) for each experimental group/condition, given as a discrete number and unit of measurement
☐	☒	A statement on whether measurements were taken from distinct samples or whether the same sample was measured repeatedly
☐	☒	The statistical test(s) used AND whether they are one- or two-sided<br>*Only common tests should be described solely by name; describe more complex techniques in the Methods section.*
☐	☒	A description of all covariates tested
☐	☒	A description of any assumptions or corrections, such as tests of normality and adjustment for multiple comparisons
☐	☒	A full description of the statistical parameters including central tendency (e.g. means) or other basic estimates (e.g. regression coefficient) AND variation (e.g. standard deviation) or associated estimates of uncertainty (e.g. confidence intervals)
☐	☒	For null hypothesis testing, the test statistic (e.g. *F*, *t*, *r*) with confidence intervals, effect sizes, degrees of freedom and *P* value noted<br>*Give P values as exact values whenever suitable.*
☐	☒	For Bayesian analysis, information on the choice of priors and Markov chain Monte Carlo settings
☒	☐	For hierarchical and complex designs, identification of the appropriate level for tests and full reporting of outcomes
☐	☒	Estimates of effect sizes (e.g. Cohen's *d*, Pearson's *r*), indicating how they were calculated

Our web collection on statistics for biologists contains articles on many of the points above.

Software and code

Policy information about availability of computer code

Data collection	Microsoft excel was used for collection of anonymised patient characteristics
Data analysis	S-LDSC v1.0.1, DESeq2 1.25.0, R-3.5.0; R-3.3.0, FeatureCounts v1.5.3, PLINK software v1.9<br>QTLtools Version 1.1, qvalue, mashR, coloc, FINEMAP v1.3

For manuscripts utilizing custom algorithms or software that are central to the research but not yet described in published literature, software must be made available to editors and reviewers. We strongly encourage code deposition in a community repository (e.g. GitHub). See the Nature Portfolio guidelines for submitting code & software for further information.

Data

Policy information about availability of data

All manuscripts must include a data availability statement. This statement should provide the following information, where applicable:

- Accession codes, unique identifiers, or web links for publicly available datasets
- A description of any restrictions on data availability
- For clinical datasets or third party data, please ensure that the statement adheres to our policy

Full summary statistics (nominal and permuted files) for data generated in this study are now available via Zenodo under accession code 10.5281/zenodo.11143561 [https://zenodo.org/records/11143561]. Expression data (cram files), and genotyping array data (plink format, .bed .bim .fam, as well as .idat and .gtc files) are shared via EGA (genotyping array data under Study Accession Number: EGAS00001002643 [https://ega-archive.org/studies/EGAS00001002643]; expression data under Study Accession Number: EGAS00001002642 [https://ega-archive.org/studies/EGAS00001002642]). Source data for the patient characteristics are provided with this paper in the accompanying Source Data file.
Previously published data used in this study can be accessed through GWAS catalog86 [https://www.ebi.ac.uk/gwas/publications/26394269;https://www.ebi.ac.uk/gwas/publications/21980299;https://www.ebi.ac.uk/gwas/publications/22057235;https://www.ebi.ac.uk/gwas/publications/24390342;https://www.ebi.ac.uk/gwas/publications/24076602;https://www.ebi.ac.uk/gwas/publications/26394269;https://www.ebi.ac.uk/gwas/publications/26502338]; GTEx Portal [https://gtexportal.org/home/datasets]; EGA [https://ega-archive.org/dacs/EGAC00001000135]; Macromap [https://zenodo.org/records/7967759]; Kim-Hellmuth et al, 2017 [http://immunpop.com/kim/eQTL]; Astle et al, 2016 [http://www.bloodcellgenetics.org]; De Lange et al, 2017 [ftp://ftp.sanger.ac.uk/pub/project/humgen/summary_statistics/human/2016-11-07/]

Research involving human participants, their data, or biological material

Policy information about studies with human participants or human data. See also policy information about sex, gender (identity/presentation), and sexual orientation and race, ethnicity and racism.

Reporting on sex and gender	Biological sex of patients included within the study is shown in Supplementary table 1.
Reporting on race, ethnicity, or other socially relevant groupings	All participants were of European ancestry
Population characteristics	Seventy-six donors were recruited; forty-two with Primary sclerosing cholangitis and ulcerative colitis and a further thirty-four with only ulcerative colitis only.
Recruitment	Donors were recruited from the autoimmune liver clinic at the Norfolk and Norwich University hospital, UK
Ethics oversight	Ethical approval was granted by the Norfolk and Norwich University Hospital (NNUH) Human Tissue Bank (reference number: 20122013-57 HT). All participants provided written informed consent.

Note that full information on the approval of the study protocol must also be provided in the manuscript.

Field-specific reporting

Please select the one below that is the best fit for your research. If you are not sure, read the appropriate sections before making your selection.

[x] Life sciences [ ] Behavioural & social sciences [ ] Ecological, evolutionary & environmental sciences

For a reference copy of the document with all sections, see nature.com/documents/nr-reporting-summary-flat.pdf

Life sciences study design

All studies must disclose on these points even when the disclosure is negative.

Sample size	Six peripheral blood cell types isolated from seventy-six individuals. No formal sample size calculation was performed, however sample size was similar to comparable eQTL studies with similar MAF filter, For example, the GTEx Consortium pilot study of post mortem tissues was able to detect tissue-specific quantitative genetic traits for a median sample size of 105 for the 9 high-priority tissues. Furthermore, the HapMap study of genetic variants underlying variation in gene expression detected an abundance of cis-regulatory variants in the human genome with a median sample size of just 40 individuals in each population group.
Data exclusions	Two individuals cell type samples were removed after failing quality control.
Replication	There were no biological replicates, however test were performed independently.
Randomization	Samples were randomly assigned to processing order, and to positions in a multi-well plates. Patient age, sex, use of drugs including 5-aminosalicylates and azathioprine, and the sequencing run were included in the model as covariates.
Blinding	The investigators were blinded to the sample group allocation when RNA sequencing and DNA genotyping was performed.

Behavioural & social sciences study design

All studies must disclose on these points even when the disclosure is negative.

Study description	
Research sample	
Sampling strategy	
Data collection	
Timing	
Data exclusions	
Non-participation	
Randomization	

Ecological, evolutionary & environmental sciences study design

All studies must disclose on these points even when the disclosure is negative.

Study description

Research sample

Sampling strategy

Data collection

Timing and spatial scale

Data exclusions

Reproducibility

Randomization

Blinding

Did the study involve field work? ☐ Yes ☐ No

Field work, collection and transport

Field conditions

Location

Access & import/export

Disturbance

Reporting for specific materials, systems and methods

We require information from authors about some types of materials, experimental systems and methods used in many studies. Here, indicate whether each material, system or method listed is relevant to your study. If you are not sure if a list item applies to your research, read the appropriate section before selecting a response.

Materials & experimental systems

n/a	Involved in the study
x	☐ Antibodies
x	☐ Eukaryotic cell lines
x	☐ Palaeontology and archaeology
x	☐ Animals and other organisms
x	☐ Clinical data
x	☐ Dual use research of concern
x	☐ Plants

Methods

n/a	Involved in the study
x	☐ ChIP-seq
☐	x Flow cytometry
x	☐ MRI-based neuroimaging

Antibodies

Antibodies used

Validation

Eukaryotic cell lines

Policy information about cell lines and Sex and Gender in Research

Cell line source(s)

Authentication

Mycoplasma contamination

Commonly misidentified lines
(See ICLAC register)

Palaeontology and Archaeology

Specimen provenance

Specimen deposition

Dating methods

☐ Tick this box to confirm that the raw and calibrated dates are available in the paper or in Supplementary Information.

Ethics oversight

Note that full information on the approval of the study protocol must also be provided in the manuscript.

Animals and other research organisms

Policy information about studies involving animals; ARRIVE guidelines recommended for reporting animal research, and Sex and Gender in Research

Laboratory animals

Wild animals

Reporting on sex

Field-collected samples

Ethics oversight

Note that full information on the approval of the study protocol must also be provided in the manuscript.

Clinical data

Policy information about clinical studies

All manuscripts should comply with the ICMJE guidelines for publication of clinical research and a completed CONSORT checklist must be included with all submissions.

Clinical trial registration

Study protocol

Data collection

Outcomes

Dual use research of concern

Policy information about dual use research of concern

Hazards

Could the accidental, deliberate or reckless misuse of agents or technologies generated in the work, or the application of information presented in the manuscript, pose a threat to:

No | Yes

- [ ] [ ] Public health
- [ ] [ ] National security
- [ ] [ ] Crops and/or livestock
- [ ] [ ] Ecosystems
- [ ] [ ] Any other significant area

Experiments of concern

Does the work involve any of these experiments of concern:

No | Yes

- [ ] [ ] Demonstrate how to render a vaccine ineffective
- [ ] [ ] Confer resistance to therapeutically useful antibiotics or antiviral agents
- [ ] [ ] Enhance the virulence of a pathogen or render a nonpathogen virulent
- [ ] [ ] Increase transmissibility of a pathogen
- [ ] [ ] Alter the host range of a pathogen
- [ ] [ ] Enable evasion of diagnostic/detection modalities
- [ ] [ ] Enable the weaponization of a biological agent or toxin
- [ ] [ ] Any other potentially harmful combination of experiments and agents

Plants

Seed stocks

Novel plant genotypes

Authentication

ChIP-seq

Data deposition

- [ ] Confirm that both raw and final processed data have been deposited in a public database such as GEO.
- [ ] Confirm that you have deposited or provided access to graph files (e.g. BED files) for the called peaks.

Data access links
May remain private before publication.

Files in database submission

Genome browser session
(e.g. UCSC)

Methodology

Replicates

Sequencing depth

Antibodies

Peak calling parameters

Data quality

Software

Flow Cytometry

Plots

Confirm that:

- [x] The axis labels state the marker and fluorochrome used (e.g. CD4-FITC).
- [x] The axis scales are clearly visible. Include numbers along axes only for bottom left plot of group (a 'group' is an analysis of identical markers).
- [x] All plots are contour plots with outliers or pseudocolor plots.
- [x] A numerical value for number of cells or percentage (with statistics) is provided.

Methodology

Sample preparation	From whole blood, peripheral blood mononuclear cells (PBMCs) were separated over Ficoll and stained them with a fluorochrome labelled antibody panel designed to isolate the six T-cell subtypes, using three rounds of two-way sorting, as shown in Source Data 2. Cells were sorted directly into chilled cell lysis buffer (Buffer RLT Plus, Qiagen) using a Sony SH800 fluorescent activated cell sorter (FACS).
Instrument	Sony SH800 fluorescent activated cell sorter
Software	FlowJo v8.0
Cell population abundance	Population abundance is show in Supplementary figure 6.
Gating strategy	Gating strategy is show in Supplementary figure 6.

- [x] Tick this box to confirm that a figure exemplifying the gating strategy is provided in the Supplementary Information.

Magnetic resonance imaging

Experimental design

Design type	
Design specifications	
Behavioral performance measures	

Imaging type(s)	
Field strength	
Sequence & imaging parameters	
Area of acquisition	

Diffusion MRI ☐ Used ☐ Not used

Preprocessing

x x

Preprocessing software	
Normalization	
Normalization template	
Noise and artifact removal	
Volume censoring	

Statistical modeling & inference

Model type and settings	
Effect(s) tested	

Specify type of analysis: ☐ Whole brain ☐ ROI-based ☐ Both

Statistic type for inference

(See Eklund et al. 2016)

Correction

Models & analysis

n/a	Involved in the study
☐	☐ Functional and/or effective connectivity
☐	☐ Graph analysis
☐	☐ Multivariate modeling or predictive analysis

Functional and/or effective connectivity

Graph analysis

Multivariate modeling and predictive analysis

This checklist template is licensed under a Creative Commons Attribution 4.0 International License, which permits use, sharing, adaptation, distribution and reproduction in any medium or format, as long as you give appropriate credit to the original author(s) and the source, provide a link to the Creative Commons license, and indicate if changes were made. The images or other third party material in this article are included in the article's Creative Commons license, unless indicated otherwise in a credit line to the material. If material is not included in the article's Creative Commons license and your intended use is not permitted by statutory regulation or exceeds the permitted use, you will need to obtain permission directly from the copyright holder. To view a copy of this license, visit http://creativecommons.org/licenses/by/4.0/
